# Unraveling the Epigenetic Role and Clinical Impact of Histone Deacetylases in Neoplasia

**DOI:** 10.3390/diagnostics11081346

**Published:** 2021-07-26

**Authors:** Dimitrios Goutas, Stamatios Theocharis, Gerasimos Tsourouflis

**Affiliations:** 1 First Department of Pathology, Medical School, University of Athens, 11527 Athens, Greece; stamtheo@med.uoa.gr; 2 Second Department of Propedeutic Surgery, Medical School, University of Athens, 11527 Athens, Greece; gtsourouflis@med.uoa.gr

**Keywords:** cancer, epigenetics, histones, biomarkers, prognosis, survival, histone deacetylase, histone deacetylase inhibitors

## Abstract

Histone deacetylases (HDACs) have long been implicated in tumorigenesis and tumor progression demonstrating their important participation in neoplasia. Therefore, numerous studies have been performed, highlighting the mechanism of HDACs action in tumor cells and demonstrating the potential role of HDAC inhibitors in the treatment of different cancer types. The outcome of these studies further delineated and strengthened the solid role that HDACs and epigenetic modifications exert in neoplasia. These results have spread promise regarding the potential use of HDACs as prospective therapeutic targets. Nevertheless, the clinical significance of HDAC expression and their use as biomarkers in cancer has not been extensively elucidated. The aim of our study is to emphasize the clinical significance of HDAC isoforms expression in different tumor types and the correlations noted between the clinicopathological parameters of tumors and patient outcomes. We further discuss the obstacles that the next generation HDAC inhibitors need to overcome, for them to become more potent.

## 1. Introduction

**Figure 1:**
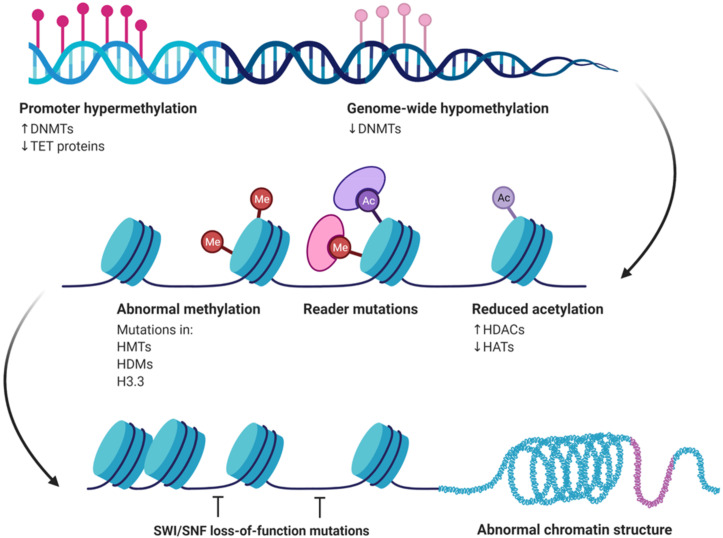
Post-translational epigenetic modifications, including methylation and acetylation, interact with gene expression either through chromatin structure alteration or histone modifiers recruitment. DNMTs: DNA methyltransferases, TET: ten-eleven translocation, HMTs: histone methyltransferases, HDMs: histone demethylases, HDACs: histone deacetylases, HATs: histone acetyltransferases, SWI/SNF: SWItch/sucrose non-fermentable, H3.3: histone variant H3.3.

Histones represent a group of proteins (H3, H4, H2A, H2B, H1), found in eukaryotic cells, that wrap around DNA and organize it into structural units, known as nucleosomes. Histones are the main protein components of chromatin and represent dynamic components in the regulation of gene transcription [1,2,3]. Various post-translational epigenetic modifications take place, including methylation, acetylation, phosphorylation and ubiquitination, interacting therefore with gene expression either through chromatin structure alteration or histone modifiers recruitment (Figure 1) [2,3].

Histone acetylation is an epigenetic modification characterized by the addition of an acetyl group to histone proteins, specifically to the lysine residues within the N-terminal tail. This histone modification is catalyzed by enzymes known as histone acetyltransferases (HATs) [1]. The two different types of HATs—cytoplasmic and nuclear—are determined based on intracellular location and histone specificity. Alternatively, histone deacetylases (HDACs) act to remove acetyl groups in a process known as histone deacetylation. Similarly, to other histone modifications, histone acetylation/deacetylation impacts chromatin structure and, in turn, gene expression, by making the DNA accessible to transcription [1]. Acetylation of lysine residues leads to a transcriptionally active chromatin structure (euchromatin) and deacetylation leads to an inactive, condensed chromatin structure (heterochromatin). Histones represent the protein backbone of chromatin, as HATs acetylate the lysine residues on histones, neutralizing their positive charge, thus shrinking their ability to bind with the negatively charged DNA [1]. Four major HDAC classes exist: Class I, Class II, Class III, and Class IV, each containing specific HDAC isoforms (Table 1) [2,3]. These classifications are based on their homology to yeast proteins. Class II HDACs are further subclassified into IIa and IIb. Class IIa consists of HDAC-4, -5, -7 and -9, all of which share a 48–57% identity [4]. Class IIb consists of HDAC-10 and -6 with homology comparison revealing a 55% overall identity and both containing a unique, putative second catalytic domain not found in other HDACs [4].

All the above could have profound effects on gene expression in normal and cancer cells [1,4,5,6,7]. 

Numerous studies have demonstrated the role of HDACs in cancer and, more specifically, in the regulation of various oncogenes; most of them have been performed by treating cancer cells with HDAC inhibitors (HDACis). Such drugs have been in the spotlight for many years since their introduction as promising therapeutic agents against cutaneous T-cell lymphoma [8]. The lead molecule used was suberoylanilide hydroxamic acid (SAHA; vorinostat) with a nanomolar affinity towards HDACs. Since then, several major classes of HDACis have been developed, including short-chain fatty-acid derived, cyclic tetra/depsipeptides, hydroxamic acid-based and amino-benzamide-based inhibitors [9], as well as a newly discovered hydrazide-based HDACi [10,11]. 

The scope of this review is to investigate the clinical significance of HDACs expression in different cancer types and their potential correlation with various clinicopathological parameters and patients’ outcomes.

## 2. Head and Neck

### 2.1. Nasopharyngeal Cancer, Salivary Glands and Oral Cancer

A notable amount of research has been performed regarding HDACs’ clinical significance in oral cancer and less in nasopharyngeal cancer and salivary gland tumors [12,13,14,15,16,17,18,19,20,21] (Table 2). HDAC-2 was found to be overexpressed in the majority of cases with epithelial dysplasia and oral squamous cell carcinoma (SCC) without, however, showing any noteworthy correlation with sex, age or oral habits in those patients [20]. Among the rest of HDACs, studied in oral cancer, HDAC-6, HDAC-8 and HDAC-9 revealed to be upregulated in oral SCC, without demonstrating any interaction with clinicopathological parameters, other than tumor aggressiveness in the case of HDAC-6 [12,13,15]. Furthermore, Theocharis et al. [21] studied the expression of HDAC-1 and HDAC-2 in mobile tongue SCC specimens, revealing a close association between their overexpression and male gender, poor histopathologic grade, positive lymph node status and depth of invasion [21].

Another study performed on a series of nasopharygeal cancer (NPC) cases, revealed a positive association among the increased HDAC-4 levels in primary and metastatic NPC tissues with poor overall survival (OS) and progression-free survival (PFS) [18]. Regarding salivary gland tumors (SGTs), the only clinical associations studied are between the expression of HDAC-1, -2, -4 and -6 in both benign and malignant tumors [19]. More precisely, HDAC-2 upregulation was associated with a better OS and prognosis, while HDAC-6 overexpression revealed to be a negative prognosticator of patients suffering from malignant SGTs [19]. Furthermore, although staining intensity did not reveal a statistical difference among benign and malignant SGTs, when coupling benign and low-grade malignant SGT cases together, the staining intensity for HDAC-2 and -6 successfully differentiated high-grade malignant tumors from benign and low-grade malignant tumors [19].

### 2.2. Thyroid

Thyroid neoplasms and their association with HDAC expression, have been poorly investigated (Table 2). The expression of HDAC -1, -2, -4 and -6 was studied in benign and malignant thyroid tissue specimens from 74 patients [22]. High HDAC-2 and HDAC-6 expression levels were more frequently observed in malignant thyroid tumors, with HDAC -1, -4 and -6 being correlated with tumor size. Additionally, HDAC-2 was correlated with lymphatic/vascular invasion, HDAC-4 with capsular invasion and HDAC -2, -4, and -6 with papillary thyroid carcinoma histotype [22].

## 3. Gastrointestinal Tract

### 3.1. Oesophagus

SCC and adenocarcinoma of the oesophagus represent the most common histologic subtypes, and efforts have been made to identify new anticancer agents that would overcome chemotherapy-resistant tumors. Among these agents HDACs have been studied for their potential application and anticancer properties [23,24,25,26] (Table 3) Huiwu et al. [26] analyzed the expression of HDAC-1, -2 and -3 in SCC oesophageal specimens and attempted to correlate their expression with clinicopathological parameters. Increased HDAC-1 but not HDAC-2 and -3 expression was noted in oesophageal SCC tissue specimens. HDAC-2 expression was associated with tumor depth of invasion, while lack of correlation between HDAC-1 and -3 with any of the clinicopathological parameters was noted [26]. Furthermore, HDAC-1, -2 and -3 showed different expression patterns, depending on ethnicity, among Kazaks and Hans. On the other hand, oesophageal adenocarcinoma was slightly associated with the expression of HDAC-2, in terms of a more aggressive tumor behavior, lacking, however, a statistically significant prognostic value [25]. Out of the Class II HDACs, only HDAC-4 has been thoroughly studied [24]. More precisely, the HDAC-4 mRNA levels from 86 patients were analyzed by real-time quantitative reverse-transcriptiase polymerase chain reaction (qRT-PCR) [24]. HDAC-4 mRNA was found to be overexpressed in all 86 cases, compared to the adjacent normal tissues, and these patients presented a shorter OS and PFS [24]. 

### 3.2. Stomach

Efforts have been made to elucidate the role of HDACs in gastric cancer [27,28,29,30,31] with, however, controversial results (Table 3). HDAC -1, -2 and -3 isoforms were examined in a cohort study of 293 patients, including 143 in the training and 150 in the validation cohort, regarding their expression levels in gastric cancer. All three isoforms were overexpressed in gastric cancer tissue specimens, while the combined overall overexpression of these HDAC isoforms was significantly associated with tumor spread and decreased patient OS [28]. In a similar fashion, Song et al. examined HDAC-2 protein expression in gastric carcinoma specimens using immunohistochemistry [27]. HDAC-2 was significantly associated with tumor aggressiveness and lymph node metastasis [27]. Out of the class II HDACs, HDAC-10 was the only member studied in a setting of clinical specimens and the results were somewhat remarkable [31]. In 179 gastric cancer tissue specimens, it was observed that HDAC-10 expression was notably decreased, in comparison to the adjacent normal tissues, and it was further correlated with patients’ gender, showing decreased expression among females, advanced disease stage, tumor invasion, nodal metastasis and tumor size [31], thereby suggesting its potential use as a prognostic marker for gastric carcinoma patients.

### 3.3. Liver

Hepatocellular carcinoma (HCC) represents the most common liver cancer entity [32]. Both class I and II HDACs aberrant expression in HCC tissues has been reported, confirming their role as chief mediators of the epigenetic mechanism of histone deacetylation [33,34,35,36,37,38,39,40] (Table 3). HDAC-1 was overexpressed in tumor tissue compared to adjacent normal liver tissue, being correlated with poor tumor grade differentiation [34]. Surprisingly, the expression of HDAC-1 in the normal hepatic tissue was linked with satellite nodules and multiple lesions, suggesting that HDAC-1 could be related to tumor spread [34]. In another study [33] direct DNA sequencing was performed in a series of 24 individuals with chronic HBV infection, identifying 22 sequence variants out of which 5 common variants were selected for genotyping. After association analyses of HBV clearance and HCC occurrence for each polymorphism and specific haplotype, one promoter polymorphism HDAC10-589C>T was revealed to be significantly associated with HCC occurrence in patients with chronic HBV infection, endorsing the importance of HDAC-10 in HCC carcinogenesis [33].

### 3.4. Pancreas and Biliary Tree

Additionally, HDACs show a strong association with pancreatic adenocarcinoma and pancreatic neuroendocrine tumors [41,42,43,44,45,46] (Table 3), the former demonstrating high chemoresistance rates, end-stage disease at the time of diagnosis in 5–28% cases, and 5-year survival of less than 5% [44,47]. Immunohistochemical analysis of class I HDACs (HDAC-1, -2, -3) demonstrated their overexpression in pancreatic adenocarcinoma tissue specimens. Nevertheless only HDAC-2 expression was correlated with poor tumor grade of differentiation [44]. On the other hand, Giaginis et al. [46] demonstrated that HDAC-2 was associated with only a few clinicopathological parameters, although in a non-significant manner, and with a marginally longer OS. Meanwhile, HDAC-1 and HDAC-4 were linked to higher proliferative capacity and HDAC-6 with an earlier histopathologic stage [46]. 

Cholangiocarcinoma (CCA), the most common malignant tumor of the biliary tree, has also shown correspondence to HDACs overexpression [48,49,50]. Immunohistochemical analysis of HDAC-3 was demonstrated to be aberrantly expressed in CCA tissues, while flow cytometry in CCA cell lines revealed that HDAC-3 overexpression induced CCA cell proliferation and inhibited apoptosis [50]. Similarly, HDAC-2, -3 and -8 were upregulated in a series of CCA specimens and, among them, HDAC-2 and HDAC-3 expression was linked to poor OS [48].

### 3.5. Colon

Surprisingly, among the four classes of HDACs, only class I HDAC has been studied in colorectal cancer regarding its clinical significance [51,52,53,54] (Table 3). Nemati et al. [51] studied HDAC3 expression in colorectal cancer (CRC) samples using RT-PCR. According to this study, enhanced HDAC-3 gene expression was noted in CRC patients, being associated with a poor grade of tumor differentiation (G3) [51]. In accordance with their results, the rest of the studies performed, also revealed the association of class I HDAC isoforms (HDAC-1, -2 and -3) with CRC, with their prevalence being linked to poor tumor differentiation, higher tumor grade and significantly reduced patient OS [52,53,54].

**Table 3:** Association of different HDAC isoforms expression in gastrointestinal tract and pancreatobiliary tumors and their correlation with clinicopathological parameters and patients’ survival.

Cancer Entity	HDAC Isoform	No. of Cases	Expression	Clinicopathologic Parameters	Survival	Method	Ref.
Oesophageal cancer	2	88 ESCC	---	Invasion depth	N/D	PCR	[26]
		132 EA	↑	Tumor aggressiveness	N/D	IHC	[25]
	4	86 ESCC	↑	Higher tumor grade	↓	PCR	[24]
Gastric cancer	1	150	↑	---	↓	IHC	[27]
	2	150	↑	---	↓	IHC	[27]
		71	↑	Advanced GC Positive LN status	↓	IHC	[27]
	10	170	↓	Advanced stage Tumor invasion Tumor size Nodal metastasis Male gender	N/D	PCR	[30]
HCC	1	156	↑	Poor tumor differentiation ↑Mortality	↓	IHC	[34]
	2	156	↑	↑Mortality	↓	IHC	[34]
	SIRT1	90	↑	Female gender AFP > 400 ng/mL P53 expression	N/D	IHC	[37]
Pancreatic cancer	1	70 PA	↑	---	↑	IHC	[46]
	4	70 PA	↑	Absence of metastasis	N/D	IHC	[46]
	6	70 PA	↑	---	↑	IHC	[46]
Cholangiocarcinoma	1	35 IHCC	↑	↑Stage LN metastasis ↓DFS LVI	↓	IHC	[49]
	2	26 CCA	↑	---	↓	IHC	[48]
	3	26 CCA	↑	---	↓	IHC	[48]
		60 CCA			↓		[50]
Colorectal carcinoma	1	140 CRC	↑	↑Tumor grade	↓	IHC	[52]
	2	140 CRC	↑	↑Tumor grade	↓	IHC	[52]
	3	140 CRC	↑	↑Tumor grade	↓	IHC	[52]
		48 CRC	↑	↑Tumor grade	N/D	PCR	[51]

ESCC: esophageal squamous cell carcinoma, EA: esophageal adenocarcinoma, SIRT1: silent mating type information regulation 2 homolog 1, PA: pancreatic adenocarcinoma, DFS: disease-free survival, IHCC: intrahepatic cholangiocarcinoma, CCA: cholangiocarcinoma, IHC: immunohistochemistry, PCR: polymerase chain reaction, AFP: alpha-fetoprotein, GC: gastric cancer, LN: lymph node, N/D: not determined, ↑: increased, ↓: decreased.

## 4. Lung

Although the role of multiple HDACis has been tested in non-small cell lung carcinoma (NSCLC), no significant research has been performed on correlations of HDAC isoforms with clinicopathological parameters and patient outcomes. Evidence from the various studies performed indicated that HDAC-1, -3, -5 and -10 were found to hold a significant role in the NSCLC progression [55,56,57,58,59,60,61] (Table 4). HDAC-1 mRNA was predominantly expressed in higher-stage (T3 or T4) lung cancer cases [55,58], and the strong HDAC-1 immunohistochemical expression (in terms of percentage and intensity) was associated with a remarkably poorer patient OS [55,58]. Meanwhile, the results regarding HDAC-5 and HDAC-10 have been somewhat controversial. More precisely, at least two studies revealed an association of HDAC-5 and HDAC-10 isoforms overexpression with higher tumor burden in NSCLC patients [59,60]. On the other hand, according to Osada et al. [57] reduced expression of both HDAC-5 and HDAC-10 proteins was associated with a poor prognosis (OS), regardless of any clinicopathologic feature, including a pathologic stage [57]. Lastly, quantification of HDAC-3 mRNA in tissue specimens of 94 lung adenocarcinoma patients revealed its correlation with clinicopathologic parameters, suggesting a significantly poorer prognosis in patients overexpressing HDAC-3 isoform [56].

## 5. Breast

In a similar fashion, the clinical role of HDACs in breast cancer, has been studied only superficially, and to a limited extent. Among them, mainly the expression levels Class I HDAC isoforms have been investigated in breast cancer and correlated with patients’ clinicopathologic parameters [62,63,64,65] (Table 4). More specifically, HDAC-2 and HDAC-3 proteins were correlated with a lower grade of tumor differentiation and negative estrogen receptor (ER) and progesterone receptor (PR) status. Additionally, HDAC-2 was also linked with cerbB2 overexpression and presence in nodal metastasis [64]. Seo et al. [62], also studied the expression of HDAC-1, -2, -3 and -6 in invasive ductal carcinoma, demonstrating similar results; HDAC-1 and HDAC-6 were associated with an improved OS in patients with ER-alpha (ERa) positive tumors. Additionally, HDAC-6 was significantly linked to ER expression and HDAC-1 to luminal type-A tumors. On the other hand, HDAC-2 and HDAC-3 were correlated with an improved OS in ER-negative tumors [62]. In accordance with that study, Zhang et al. [63] examined HDAC-6 mRNA expression levels in 135 female patients with invasive breast carcinoma, concluding that HDAC-6 was predominantly expressed in patients with small tumors (<2 cm), of low histologic grade, presenting positive hormone-receptor status [63]. Based on their study, HDAC-6 overexpression in breast cancer should be related with an improved patients’ OS.

**Table 4:** Association of different HDAC isoforms expression in lung and breast tumors and their correlation with clinicopathological parameters and patients’ survival.

Cancer Entity	HDAC Isoform	No. of Cases	Expression	Clinicopathologic Parameters	Survival	Method	Ref.
Lung carcinoma	1	93 LA	↑	↓5 year-DFS	↑	PCR	[55]
		102 NSCLC	↑	↑Tumor stage		PCR	[58]
	3	94 LA	↑	↓5 year-DFS	↓	PCR	[56]
	10	180 NSCLC	↑	---	↓	IHC	[61]
		74 NSCLC	↓	---	↓	PCR	[57]
Breast carcinoma	1	238	↑	HR (+)	N/D	IHC	[64]
					N/D	IHC	[62]
		300 IDC	↑	LumA tumors	N/D	IHC	[64]
	2	238	↑	Poor differentiation HR (−) ↑CerbB2	N/D	Genomic analyses	[65]
	3	3000	↑	↑Tumor grade Positive LN	↓	IHC	[64]
	5	238	↑	Poor tumor differentiation HR (−)	N/D	IHC	[62]
	6	300 IDC	↑	ER (+) LumB tumors	N/D	IHC	[63]
		135	↑	↑ DFS ER (+) PR (+) Tumor size <2 cm Low histologic grade ↑Response in endocrine treatment	N/D	qRT/PCR	

LA: lung adenocarcinoma, NSCLC: non-small cell lung carcinoma, HR: hormone receptors, IDC: invasive ductal carcinoma, LumA: luminal A, LN: lymph node, DFS: disease-free survival, LumB: luminal B, ER: estrogen receptor, PR: progesterone receptor, IHC: immunohistochemistry, qRT-PCR: real-time quantitative reverse-transcription polymerase chain reaction, PCR: polymerase chain reaction, N/D: not determined, ↑: increased, ↓: decreased.

## 6. Urogenital Tract

### 6.1. Kidney

HDACs have been associated with normal kidney development at the level of differentiation and maintenance of nephron progenitor and, meanwhile, they have constantly been linked with the development of numerous kidney diseases [66,67,68]. HDAC-1 and HDAC-6 are among the most well-studied HDACs, being related with kidney cancer initiation, progression and metastasis [69,70,71] (Table 5). Immunohistochemical analysis for HDAC-1 on tissue microarray (TMA) specimens of clear-cell renal-cell carcinoma (ccRCC) patients, revealed direct association of HDAC-1 overexpression with positive hypoxia inducible factor (HIF) isoforms (predominantly HIF1a/HIF2a) expression [71]. Nevertheless, no significant association of HDAC-1 expression with either a tumor’s histopathologic grade, OS or disease-specific survival was noted. However, according to The Cancer Genome Atlas (TCGA) data-set, 4% of patients with ccRCC show upregulation of HDAC-1 and HDAC-6 mRNA and direct association with an advanced tumor stage in a, nevertheless, insignificant manner [71,72]. Furthermore, overexpression of HDAC-6 was noted in patients with ccRCC, alongside concomitant expression of ERa regardless of the patient’s gender. Their analysis revealed that both HDAC-6 and ERa were predominantly expressed in the cytoplasm, exhibiting an overall better clinical response, similar to that of patients with breast tumors receiving tamoxifen [71]. HDAC-2 has also been associated with renal-cell carcinoma, both in the clear-cell and papillary variants but also in chromophobe RCCs [70]. Contrary to the expression of HDAC-1 and -2 in RCCs, HDAC-3 is significantly less expressed in ccRCCs and papillary RCCs, and remains completely negative in chromophobe RCCs [70]. Nevertheless, according to this study no significant correlation between HDAC-1, -2 and -3 and a patients’ age, histological tumor grade or TNM status was noted [70]. The above-mentioned associations of HDACs with other factors could serve for combinatorial optimization of new potential therapeutic targets; for example, the combination of tamoxifen with an HDAC-6 inhibitor for patients with ER positive HDAC-6 expressing kidney tumors [71]. 

### 6.2. Urinary Bladder

Based on the research performed so far, the role of HDACs in bladder cancer remains controversial, most probably due to the significant intertumoral heterogeneity at both a molecular and phenotypic level. Class I HDACs have shown to be overexpressed in bladder cancer [73,74,75,76] (Table 5). Immunohistochemical analysis of clinical samples obtained from radical cystectomy and transurethral bladder tumor resection (TURBT) specimens revealed high expression of HDAC-1, -2 and -3 in 40 to 60% of all tumors [73]. The expression of all three HDACs in tumor cells was strongly correlated with high-grade urothelial carcinoma. Additionally, HDAC-2 expression in the neoplasm, was also shown to be associated with increased incidence of a carcinoma in situ component adjacent to the invasive carcinoma [73]. In another study, it was reported that although HDAC-1 mRNA levels were significantly elevated in bladder cancer tissue samples, no associations with clinicopathologic characteristics such as age, gender, muscle invasion or histological grade was noted [76]. Similarly, HDAC-6, a class II HDAC, although overexpressed in high-grade urothelial carcinomas, showing predominantly a cytoplasmic staining pattern, and remaining completely negative in low grade tumors, failed to associate with any of the clinicopathological characteristics examined [77].

### 6.3. Prostate

A few studies performed on prostate cancer revealed a direct association of both class I and class II HDACs with prostate carcinoma, and in certain cases, with Gleason’s score [78,79,80] (Table 5). Overexpression of HDAC-1, -2 and -3 was reported in most prostate carcinoma cases. HDAC-1 and HDAC-2 were mostly expressed in prostate cancer tissues of patients with Gleason score >7, while HDAC-3 failed to associate with Gleason’s score, [80]. Furthermore, HDAC-1, HDAC-2 and HDAC-3 expression levels were positively correlated with tumor proliferative status, assessed as Ki67 labeling index. HDAC-2 expression was further associated with a higher relapse-free survival [80]. Among class II HDACs, HDAC-4 and HDAC-5 were significantly overexpressed in prostate cancer tissues and even more in the cases of recurrent prostate cancer [78].

### 6.4. Testis

Little is known regarding HDAC expression and testicular cancer (Table 5). Fritzsche et al. [81] analyzed the expression of class I HDAC isoforms (HDAC-1, -2 and -3) in a subset of 325 testicular germ-cell tumors through immunohistochemistry. HDAC-2 and HDAC-3 were shown to be overexpressed regardless of histological subtype, while HDAC-1 was consistently expressed in lower levels. On the contrary, choriocarcinoma tumor samples showed to express all the above class I HDAC isoforms (HDAC-1, -2, -3) at high levels [81]. 

## 7. Female Genital Tract

Among the various cancer types that can develop along the female genital tract, it has been observed that HDACs can play a pivotal role in their development, either in promoting carcinogenesis [82,83,84,85,86,87,88] or in certain cases acting as a tumor suppressor [89] (Table 5). Class I HDAC isoforms showed to be overexpressed in all histologic subtypes of tumors arising both in the endometrium and in the ovaries [82,83]. However, HDAC-2 demonstrated the highest rates of expression in endometrial and ovarian cancer and serous and clear-cell subtypes the most frequent endometrioid adenocarcinomas where all HDAC isoforms were overexpressed [82]. Yano et al. [87] studied the expression of HDAC-1, -2, -3, -4, -5, -6 and -7 in 201 ovarian cancer tissue specimens of all subtypes, including post-chemotherapy samples. According to this study, the above HDACs not only were overexpressed in all histologic subtypes of ovarian cancer, but also proved to affect prognosis and chemotherapy response. More specifically, HDAC-6 and HDAC-7 overexpression was correlated with a poor prognosis in clear cell carcinoma, while HDAC-1 was associated with decreased OS in serous carcinomas [87]. Furthermore, HDAC-1, -6 and -7 were upregulated following chemotherapy, therefore constituting a possible adjunct target [87]. In a similar fashion, class I HDAC isoforms, proved an upregulation in vulvar neoplasia, and more precisely in vulvar intraepithelial neoplasia (VIN) and vulvar squamous cell carcinoma (VSCC) [85]. HDAC-2 was significantly increased in VIN in comparison to VSCC, while HDAC-3 levels were higher in VSCC rather than in VIN. On the other hand, HDAC-1 levels did not show any significant difference in expression levels among VIN and VSCC [85]. Conversely, Chenlin et al. [89] demonstrated that HDAC-10 could act as a tumor suppressor in cervical cancer tissue specimens and suppress lymph node metastasis mostly suppressing the expression of matrix metalloproteinase (MMP) -2 and -9 genes [89], therefore constituting a potential therapeutic target for cervical cancer.

**Table 5:** Association of different HDAC isoforms expression in urogenital tract tumors and their correlation with clinicopathological parameters and patients’ survival.

Cancer Entity	HDAC Isoform	No. of Cases	Expression	Clinicopathologic Parameters	Survival	Method	Ref.
Urinary bladder cancer	1	174 BC	↑	↑Tumor grade	N/D	IHC	[69]
				↓Prognosis			
	2	174 BC	↑	↑Tumor grade		IHC	[69]
Prostate cancer	1 2	192 192	↑	↑Gleason score	N/D	IHC IHC	[76] [76]
				↑Gleason score			
			↑	↑DFS			
Female genital tract	1	115 OC	↑	---	↓	IHC	[88]
		465 OEC 149 EC 22 OC	↑ ↑ ↑	↓DFS ↓DFS ↑Stage		IHC IHC IHC	[82] [82] [86]
		201 OC	↑	----	↓ in SEC and EC	IHC	[87]
	2	59 VSCC 465 OEC 149 EC 22 OC	↑ ↑ ↑ ↑	↑Tumor stage ↓DFS ↓DFS ↑Tumor stage	N/D	IHC IHC IHC IHC	[85] [82] [82] [86]
	3	465 OEC 149 EC	↑ ↑	↓DFS ↓DFS	N/D	IHC IHC	[85] [82]
	6	201 OC	↑	---	N/D	IHC	[85]
	7	201 OC	↑	---	N/D	IHC	[82]
	10	60 CC	↑	↓Tumor stage Absent LN metastasis	↓	IHC	[87] [89]

BC: bladder cancer, OC: ovarian cancer, OEC: ovarian endometrioid carcinoma, EC: endometrioid carcinoma, VSCC: vulvar squamous cell carcinoma, SEC: serous carcinoma, IHC: immunohistochemistry, DFS: disease-free survival, CC: cervical cancer, N/D: not determined, ↑: increased, ↓: decreased.

## 8. Melanoma

Limited data exist regarding HDAC status and melanoma. HDAC-3, -5, -6 and -8 have been associated with primary and metastatic melanoma cases [90,91] (Table 6). Liu et al. [90] revealed an association among HDAC-5 and -6 upregulation and primary melanoma tissue specimens compared to the adjacent normal skin, without, however, revealing any further association with clinicopathologic parameters. On the other hand, Wilmott et al. [91] demonstrated that HDAC-3 nuclear expression was associated with a better prognosis, while HDAC-8 cytoplasmic overexpression is directly linked with an increased OS in patients with stage IV metastatic melanoma and with positive *BRAF/NRAS* mutation status [91].

## 9. Mesenchymal Tumors

The role of epigenetics in mesenchymal neoplasia has not been thoroughly studied, with only a limited amount of research performed [84,92,93,94] (Table 6). Que et al. [94] analyzed the expression patterns of class I HDACs performing RT-PCR in human soft-tissue sarcoma samples. Patients with HDAC-1 and HDAC-2 higher expression levels presented significantly lower OS rate [94]. Furthermore, a different study performed in osteosarcoma specimens revealed HDAC-2 as the most frequently upregulated isoform. Additionally, low expression levels of HDAC-1 were associated with late Enneking stages, a metastatic phenotype, and also serve as a significant negative predictor of high-grade osteosarcomas [92]. Finally, Pacheco and O Nielsen [93] tried to elucidate the role of HDAC-1 and HDAC-2 in a large series of mesenchymal tumors, comprised of 1332 cases, representing 44 categories of both malignant and borderline tumors. According to their study, HDAC-2 was the most frequently expressed isoform in translocation-associated sarcomas in comparison to the other mesenchymal tumors. Additionally, HDAC-1 was the least frequently expressed isoform in comparison to the rest of mesenchymal tumors [93]. Such results indicate that HDAC-2 is the most probable isoform in contributing to the pathogenesis of translocation-associated mesenchymal tumors and, therefore, a possible therapeutic target.

## 10. Neuroendocrine Neoplasms

Pancreatic neuroendocrine tumors (pNETs) represent the only category of neuroendocrine neoplasms studied for their clinical association with HDACs expression [42,43] (Table 6). More precisely, Klieser et al. [42] analyzed the expression patterns of all HDAC classes in TMAs, representing 57 pNET cases comprised of different grades and TNM stages. Among all HDAC classes, although all isoforms were upregulated, HDAC-5 was revealed to share the highest prognostic index. More specifically, HDAC-5 upregulation was linked with metastasis and a poor OS for patients with pNETs. On the other hand, members of the HDAC classes I, III, and IV were correlated with increased proliferation index (Ki67 labelling index) [42]. Furthermore, HDAC-3 and HDAC-4 showed aberrant expression and close relation to miRNA449a, although of none clinical significance [43]. 

## 11. Brain Tumors

Limited data regarding the clinical significance of HDACs and their possible contribution to brain neoplasia exist in the literature [95,96] (Table 6). Lucio-Eterovic et al. [95] compared mRNA and protein levels of 12 HDAC genes (class I, II and IV HDACs) in 43 tumor samples (20 were low-grade gliomas and 23 were high-grade gliomas). Their study revealed decreased mRNA expression levels of Class II and IV HDACs in glioblastomas and grade III astrocytomas, when compared to the expression levels in low-grade gliomas [95]. This negative correlation pattern between HDAC gene expression and low-grade gliomas raises the question on whether an HDACi would be of any value in this devastating disease. On the other hand, gene expression arrays performed in medulloblastoma primary samples, using qRT-PCR and IHC, for the detection of HDAC-1 through HDAC-11 expression levels revealed that HDAC-5 and HDAC-9 isoforms were constantly overexpressed compared to the adjacent non-neoplastic brain tissue [96]. Furthermore, these isoforms were positively correlated with high-risk medulloblastomas and with poor survival, signifying their role as valuable markers in risk stratification [96]. 

**Table 6:** Association of different HDAC isoforms expression in other types of tumors and their correlation with clinicopathological parameters and patients’ survival.

Cancer Entity	HDAC Isoform	No. of Cases	Expression	Clinicopathologic Parameters	Survival	Method	Ref.
Melanoma	3	175 Stage IV melanomas	↑	---	↑in nuclear expression	IHC	[91]
	8	175 Stage IV melanomas	↑	---	↑in cytoplasmic expression	IHC	[91]
Mesenchymal tumors	1	89 Osteosarcomas	↓	Metastases ↑Tumor stage	↓	IHC	[92]
		49 STS	↑	---	↓	RT-PCR	[93]
	2	89 Osteosarcomas	↓	---	↓	IHC	[92]
		49 STS	↑	---	N/D	RT-PCR	[93]
	3	89 Osteosarcomas	↓	Age >15 y.o	↓	IHC	[92]
Neuroendocrine tumors	5	57 pNETs	↑	Metastasis	↓	IHC	[42,43]
Brain tumors	5	140 Medulloblastomas	↑	↑Tumor grade	↓	IHC, qRT-PCR	[96]
	9	140 Medulloblastomas	↑	↑Tumor grade	↓	IHC, qRT-PCR	[96]
		43 Glioblastomas	↓	↑Tumor grade	N/D	IHC, qRT-PCR	[95]

STS: soft-tissue sarcomas, IHC: immunohistochemistry, qRT-PCR: real-time quantitative reverse-transcription polymerase chain reaction, PCR: polymerase chain reaction, pNETs: pancreatic neuroendocrine tumors, N/D: not determined, ↑: increased, ↓: decreased.

## 12. Conclusions

Among the four different HDAC classes, classes I and II seemed to be more frequently correlated with tumor aggressiveness, in terms of grade, stage, size and nodal metastasis, as well as, with OS and disease-free survival (DFS). Most of the times HDACs were upregulated, leading to a more aggressive tumor phenotype, while in few cases they were downregulated and, also, associated with a poor patient prognosis. Nevertheless, the close association of various HDAC isoforms with neoplasia in general and their direct correlation with tumors’ clinicopathological aspects and survival data, highlights their potential significance as prognostic biomarkers and as potent therapeutic target agents. Immunohistochemistry and qRT-PCR could serve as valuable assets in identification and quantification of the HDAC isoforms overexpressed or downregulated in each individual patient and, thereby, assist to evaluate their prognosis. These data could further serve in developing a personalized treatment approach for every individual, which would be based on the levels and the type of isoform expressed. Nevertheless, despite the undeniable efficacy of HDACis in hematological malignancies [97], their use as monotherapy in solid tumors has failed to provide any promising results, partly due to their serious side effects, and partly due to the negligible number of patients in phase II clinical trials that reached a complete or partial response [98,99,100,101,102,103,104,105]. However, combination of HDACis with other chemotherapeutic agents in solid tumors have shown notable results. More specifically, the tripartite therapy of belinostat, carboplatin and paclitaxel in pretreated patients with epithelial ovarian cancer was well tolerated and revealed a clinical benefit [104]. A precise explanation on the mechanism responsible for the higher efficacy of HDACis in hematological malignancies is lacking. Possibly their poor pharmacokinetics could explain the reason why they fail to reach therapeutic concentrations, due to their short half-life (time) in vivo [106,107].

The successful application of HDACis in the field of cancer will rely heavily on the improvement of in vivo pharmacokinetic properties of the next generation HDACis and on the improvement of their potency and selectivity. Selective targeting of specific HDAC isoforms could potentially increase their efficacy and decrease their toxic effects. Nevertheless, as they display unique cellular toxicity profiles, greater knowledge regarding HDAC biology in neoplasia and discovering new biomarkers that could predict therapeutic effects would allow the detection of those individuals that would mostly benefit from the therapy with HDACis.

## Figures and Tables

**Table 1:** HDAC classification.

Group	Class	Isoform
Zn dependent	*Class I*	HDAC1
		HDAC2
		HDAC3
		HDAC8
	*Class IIa*	HDAC4
		HDAC5
		HDAC7
		HDAC9
	*Class IIb*	HDAC6
		HDAC10
	*Class IV*	HDAC11
NAD dependent	*Class III*	SIRT (1–7)

**Table 2:** Association of different HDAC isoforms expression in head and neck tumors and their correlation with clinicopathological parameters and patients’ survival.

Cancer Entity	HDAC Isoform	No. of Cases	Expression	Clinicopathologic Parameters	Survival	Method	Ref.
Oral cancer	2	93 OSCC		---	↓	IHC	[20]
		49 TSCC		Muscular invasion	N/D		[21]
	6	90 OSCC	↑	Tumor stage	N/D	IHC, PCR	[12]
	9	60 OSCC	↑	---	↓	PCR	[15]
Salivary gland tumors	2	22	↑	---	↑	IHC	[19]
	6			Tumor aggressiveness	↓		
Thyroid cancer	1	47	↑	↑Tumor size	N/D	IHC	[22]
	2			LVI	N/D		
	4			↑Tumor size and Capsular invasion	N/D		
	6			↑Tumor size	N/D		
Nasopharyngeal cancer	4	74	↑	Shorter PFS	↓	IHC, PCR	[18]

OSCC: oral squamous cell carcinoma, TSCC: tongue squamous cell carcinoma, OS: overall survival, IHC: immunohistochemistry, PCR: polymerase chain reaction, LVI: lymphovascular invasion, PFS: progression free survival, IHC: immunohistochemistry, N/D: not determined, ↑: increased, ↓: decreased.
